# A Framework for Different Levels of Integration of Computational Models Into Web-Based Virtual Patients

**DOI:** 10.2196/jmir.2593

**Published:** 2014-01-23

**Authors:** Andrzej A Kononowicz, Andrew J Narracott, Simone Manini, Martin J Bayley, Patricia V Lawford, Keith McCormack, Nabil Zary

**Affiliations:** ^1^Digital Patient LabDepartment of Learning, Informatics, Management and EthicsKarolinska InstitutetStockholmSweden; ^2^Department of Bioinformatics and TelemedicineFaculty of MedicineJagiellonian UniversityKrakówPoland; ^3^Medical Physics GroupDepartment of Cardiovascular Science, Faculty of Medicine, Dentistry and HealthUniversity of SheffieldSheffieldUnited Kingdom; ^4^INSIGNEO Institute for in silico MedicineUniversity of SheffieldSheffieldUnited Kingdom; ^5^Department of Biomedical EngineeringIRCSS Mario Negri InstituteBergamoItaly; ^6^Orobix SrlBergamoItaly

**Keywords:** computer simulation, computer-assisted instruction, education, medical, medical informatics applications

## Abstract

**Background:**

Virtual patients are increasingly common tools used in health care education to foster learning of clinical reasoning skills. One potential way to expand their functionality is to augment virtual patients’ interactivity by enriching them with computational models of physiological and pathological processes.

**Objective:**

The primary goal of this paper was to propose a conceptual framework for the integration of computational models within virtual patients, with particular focus on (1) characteristics to be addressed while preparing the integration, (2) the extent of the integration, (3) strategies to achieve integration, and (4) methods for evaluating the feasibility of integration. An additional goal was to pilot the first investigation of changing framework variables on altering perceptions of integration.

**Methods:**

The framework was constructed using an iterative process informed by Soft System Methodology. The Virtual Physiological Human (VPH) initiative has been used as a source of new computational models. The technical challenges associated with development of virtual patients enhanced by computational models are discussed from the perspectives of a number of different stakeholders. Concrete design and evaluation steps are discussed in the context of an exemplar virtual patient employing the results of the VPH ARCH project, as well as improvements for future iterations.

**Results:**

The proposed framework consists of four main elements. The first element is a list of feasibility features characterizing the integration process from three perspectives: the computational modelling researcher, the health care educationalist, and the virtual patient system developer. The second element included three integration levels: basic, where a single set of simulation outcomes is generated for specific nodes in the activity graph; intermediate, involving pre-generation of simulation datasets over a range of input parameters; advanced, including dynamic solution of the model. The third element is the description of four integration strategies, and the last element consisted of evaluation profiles specifying the relevant feasibility features and acceptance thresholds for specific purposes. The group of experts who evaluated the virtual patient exemplar found higher integration more interesting, but at the same time they were more concerned with the validity of the result. The observed differences were not statistically significant.

**Conclusions:**

This paper outlines a framework for the integration of computational models into virtual patients. The opportunities and challenges of model exploitation are discussed from a number of user perspectives, considering different levels of model integration. The long-term aim for future research is to isolate the most crucial factors in the framework and to determine their influence on the integration outcome.

## Introduction

### Background

Computers and Internet technologies have already entered the mainstream of health care education [1]. Although a gap still exists between what is technically possible in pilot studies and the realities of educational practice, we have reached a stage where Web-based training is regarded as routine [2,3]. The use of such technology is driven not only by the urge for innovation, a willingness to improve teaching quality, and limited personnel, but also by cost-effectiveness analysis [4].

The use of virtual patients is undeniably one of the techniques most often associated with application of computer-aided training in health care [2,5] but, as is often the case with new concepts, the understanding of the term “virtual patient” varies depending on the research community. We define a virtual patient as “interactive computer simulation of real-life clinical scenarios for the purpose of health care and medical training, education or assessment” [6]. This excludes other methods used in medical education such as human role-playing, computerized mannequins, part-task trainers, and systems requiring specialized equipment [7], as well as all non-educational virtual patients.

While complex, immersive virtual reality scenarios are technologically possible (eg, [8]), the routine use of virtual patients often focuses on technically simple solutions. Huwendiek et al attempted to classify this type of virtual patient solution with four example systems: CAMPUS, CASUS, Open Labyrinth, and Web-SP [9]. While these systems differ in many respects, they have in common the presentation of a clinical, case-based scenario divided into discrete steps displayed either (1) linearly, with a single final outcome, or (2) branched, enabling different narration paths depending on user choice. All these systems are Web-based and simply require an Internet connection through a standard browser. By common agreement, the ANSI-accredited MedBiquitous Virtual Patient (MVP) standard [10] has been adopted by the medical education community for the representation of virtual patient data. Implementation of support for the standard in four systems was achieved as part of the eViP project [11].

There are at least two significant advantages of this type of virtual patient. The first is the possibility of significant teacher involvement in the development process. This has been enabled by investment in the user-friendliness of authoring tools and the simplification of technical workflows to enable medical experts to focus on the content of virtual patients. As a result, virtual patients of this class are generally tailored to the needs of a particular teacher and institution, to the type of educational activity, and to the specific learning objectives. This might not be the case with technologically complex virtual patients. The second advantage is the high level of accessibility of this type of virtual patient to learners over the Internet with personal computers, or even “just-in-time” access with mobile devices. Accessibility is also enhanced by the low cost of licensing of these virtual patient systems.

There are several possible “next steps” in the development of virtual patients from the class described above. Our chosen step is to increase the interactivity of virtual patients by enriching them with computational models of physiological and pathological processes. Here we use the term “computational model” following the definition given by Garrido [12] as a “mathematical model implemented in a computer system that requires high performance computational resources to execute”. This usually requires the numerical solution of non-linear models. Being cognizant of the long research tradition of modeling and simulation in biomedical engineering (eg, [13]), it is not our intention to create new models but rather to seek opportunities to integrate existing models into present virtual patient systems.

Here we propose a framework to support integration of existing computational models of physiological function within virtual patients. This framework is expected to define concepts that help in formalizing the integration and evaluating the feasibility of the integration. Extending virtual patients with computational models is recognized as a promising trend in the current virtual patient literature (eg, [3,14]).

While integration frameworks for simulation in health care education have been published previously (eg, [15-17]), the focus of these earlier publications is different from that envisaged here. The “Practica continua” framework, developed by Ellaway et al [15], discusses integration of a broad range of simulation modalities, covering methods such as human role-playing (eg, standardized patients), physical equipment (eg, computerized mannequins, part-body task trainers, and haptic devices), teleconferencing, and 3D visualization tools. The framework we present is focused solely on integration of physiological computational models derived from existing research projects, such as the Virtual Physiological Human (VPH) initiative [18], within screen-based, narrative virtual patient systems (as used in the eViP project [9]).

The RICORDO framework has a more technical scope, with the aim of improving the accessibility of simulation data and model resources for physiology and pharmacology research, as well as health care education [16]. This involves selection of technical standards for the use of controlled vocabularies, ontologies, and metadata encoding representation. In addition, an open toolkit for creation of ontology composites and annotation of resources was developed. A critical appraisal of technical standards for sharing educational resources in medical education was also undertaken by the mEducator Best Practice Network [19]. These contributions can complement the methods described in the current paper by facilitating the discovery of new models to be integrated in existing virtual patients and by promoting dissemination of these based on clinical classification of the content.

Finally, frameworks are described within the literature, which facilitate the integration of various software libraries for high-fidelity medical simulation. For instance, Halic et al proposed a framework called SoFMIS for rendering 3D scenes in surgical simulation involving the use of haptic devices [17]. A related framework adopts the SoFMIS architecture for simulation in Web environments [20]. In contrast to this approach, our framework deals more with virtual patient authoring strategies than with concrete software solutions. The SoFMIS framework demonstrates how different software libraries may be grouped in modules supporting reusability. We intend to extend the existing, well-established systems with new elements. Furthermore, our virtual patients are directed towards improving clinical reasoning skills (as diagnostic and treatment selection processes) and do not focus on the psychomotor and procedural skills that would be required in high-fidelity surgical simulations.

### Objective

The aim of this paper is to propose a conceptual framework to organize research and development towards the integration of computational models within virtual patients. In particular, we focus on (1) characteristics to be addressed while preparing the integration, (2) the extent of the integration, (3) strategies to achieve integration, and (4) methods for evaluating the feasibility of integration.

The framework will be considered successful if its application leads to practical recommendations and predictions enabling the medical education and simulation communities to collaborate in the integration of computational models within virtual patients. Our long-term aim is to isolate the crucial factors and determine their influences on the success of the integration process in supporting the practical use of the virtual patient for teaching. One way of achieving this is to evaluate the reaction of a suitable target audience to virtual patients implemented with different levels of model integration proposed by the framework. For this reason, an additional goal is to pilot an investigation of altered perceptions of virtual patients at two levels of integration.

## Methods

### Process for Developing the Framework

The framework will be initiated using an iterative process inspired by the Soft System Methodology (SSM) [21]. The SSM method was designed to aid analysis of situations involving groups with interacting perceptions where human-related aspects play an important role. The methodology looks for solutions that are both desirable and feasible considering the components of the system.

While developing virtual patients, we should address human-related aspects. For instance, their construction should be influenced by actual learning needs and preferences of learners and not by the accuracy of simulation results alone. This makes SSM methodology particularly suitable for the purpose of developing the framework. This methodology has been previously applied to design conceptual frameworks to introduce information technologies into health care, for instance by Ruotsalainen et al to define a framework for Trusted Pervasive Health [22].

The SSM method consists of four steps, constituting a learning cycle, which are repeated to improve the system design [21]. This involves (SSM Step 1) characterization of the system in question including description of its features, problems of interest, risks, and challenges, (SSM Step 2) expression of the ideas and planned actions in the form of models encapsulating different perceptions, (SSM Step 3) testing of the model on a real-world example, and (SSM Step 4) synthesis of the system description with verification outcomes to propose improvements for the next development cycle.

### Source of Computational Models

The initial framework design is informed by experience acquired by the authors in previous projects dealing with virtual patients, health care education and biomedical modeling simulation, including eViP [6] and the Virtual Physiological Human (VPH) initiative [18]. The recently established collaboration between the VPH Network of Excellence (NoE) and representatives of the Association for Medical Education in Europe (AMEE) health care educational community provided an appropriate environment to develop the framework.

The overarching aim of the VPH is to establish methods and tools for computational analysis of the human body, integrating the diverse nature of physiology and pathophysiology of the different organ systems [18]. The VPH combines expertise in computer modeling and clinical research to deliver a spectrum of advanced simulations of physiological function, disease development and progression, and response to intervention. The ambitious goal is to develop a technological infrastructure based on patient-specific data to deliver prediction of clinical outcomes by means of quantitative models that integrate biophysical processes across diverse scales from the molecular level, to organ systems, and even populations [23]. The clinical targets of VPH projects are diverse and address the challenges of computational modeling in cardiology (eg, euHeart), vascular pathology (eg, ARCH), or various types of cancer (eg, PASSPORT) [24]. Effort has also been allocated to address the technical aspects of the computational infrastructure required by VPH projects (eg, VPH Share).

The VPH NoE [25] has provided a central focus for VPH project output, developing best practice and support for the exposure and sustainability of VPH-related tools, training, and standards. An important task of the VPH NoE has been to ensure that the academic, medical, and industrial domains have access to a workforce that is well prepared to meet the possibilities offered by the VPH. This has been addressed through delivery of training activities (workshops, summer schools) and the development of educational materials to raise awareness of these new technologies [24]. The educational goals of VPH NoE align well with the aims of the virtual patient community for more interactive virtual patients.

### Demonstration of the Framework

#### Selected Model for Integration

The proposed framework is showcased using a real-world example: the VPH ARCH project [26]. This project developed vascular access modeling for surgical planning for hemodialysis in end-stage renal disease (ESRD) patients. While there are several methods to create vascular access for ESRD patients, the arteriovenous fistula (AVF) is preferred due to reduced potential complications. An AVF may be created either in the lower arm (radiocephalic fistula) or upper arm (brachiocephalic or brachiobasilic fistula) [27]. Deciding where to create the fistula is informed by preoperative physical examination and duplex ultrasound evaluation of the vasculature of the arm. Within the VPH ARCH project a computational model was developed to support clinical decision making. The simulation is based on a distributed lumped-parameter implementation of a wave propagation model [28], which has been evaluated with clinical data. The computational model is available under an open-source license as part of a software toolkit called archTk [29] and as part of a clinical Web application [30].

#### The Integration Process

**Figure 1:**
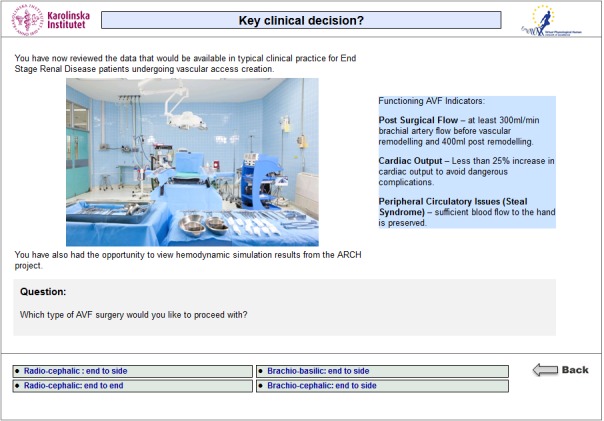
The virtual patient case containing archTk simulation outcome wrapped in a stand-alone, Web-enabled virtual patient player.

Using the archTk model and a set of patient input parameters, we simulated blood flow and pressure in the upper extremity after implementing different types of AVF, incorporating simulation outcomes into a virtual patient. The case presents the story of John Jones: a 68-year-old male who was diagnosed with chronic kidney disease following an infection. The virtual patient was developed under the supervision of clinical experts by authors of this paper. The basic version contains 28 screen cards connected in a graph structure to provide branching possibilities (Figure 1). The screen cards comprise narratives, medical examination results (including simulation outcomes), and images. The integration was performed using Bit Pathways [31,32]. This authoring tool was developed by one of the paper’s authors and allows export of the graph structure, including the case data, as an MVP package [10]. Such reusable learning objects are readable by all MVP-supported virtual patient systems. Alternatively, the content may be wrapped by the Bit Pathways authoring tool into an HTML/Java Script player to form a stand-alone or Web-based application (Figure 1).

### Evaluation Event

#### Context Description

To test the potential of the proposed framework as a tool for discriminating between different levels of simulation integration, a virtual patient exposing the archTk simulation results was prepared in two variants, reflecting the different integration levels defined by the framework. Both variants of the case were demonstrated during a dissemination and evaluation workshop organized by the VPH NoE at the European Vascular Course (EVC) conference in Maastricht in March 2013. Access to the virtual patients was offered to the participants by 10 Internet-connected Apple iPad 2 tablets. The virtual patient variants were stored on a remote Web server. The user interface of the Web application was tailored to the size and navigation possibilities of the tablet’s touch screen. Participants were allocated to one of the two virtual patient variants at random (Study Groups 1 and 2) based on the tablet the user selected to sit at in the workshop. Participants were blinded to this selection to prevent bias (Hawthorne effect).

Participation in the workshop was voluntary. Participants were recruited by email announcements broadcast by the conference organizers, posters at the conference venue, and direct invitations with pamphlets handed out during coffee breaks. Participation required registration for a specific workshop session due to a limit of 10 individuals per session. Use of the virtual patient was preceded by a 15-minute introduction to the VPH initiative and virtual patient tools. A Web-based evaluation questionnaire, authored with Google Forms, was completed immediately after the session. User identification was based only on the tablet ID and time of questionnaire completion. The questions asked pertained to the level of interest in the virtual patient and agreement with the content. In addition, the study group dealing with the higher integration level were given the possibility of commenting on the added interactivity (as explained in the results section of this paper).

#### Study Participants

Thirty-eight participants filled in the questionnaire. The distribution of the participants in study groups was well balanced with 20 (53%) in Study Group 1 and 18 (47%) in Study Group 2. Basic demographic and background data are presented in Table 1. Most of the participants were clinicians specializing in vascular surgery or nephrology (28/38, 74%). The remaining participants were nurses, biomedical engineers, and producers of medical equipment (9/38, 24%; 1 missing response).

**Table 1:** Summary of the study participants’ demographic data.

	Group 1	Group 2
Number of participants	20	18
Gender: female/male/no answer, %	75/25/–	61/22/17
Age, yrs, mean (SD)	41.6 (12.6)	36.0 (11.8)
Physician, %	85	61
Work experience, yrs, mean (SD)	11.8 (12.5)	9.5 (9.0)
Involved in teaching, %	80	72

#### Statistical Analysis

Differences between the two groups were assessed using a non-parametric double-sided Mann-Whitney U-test. The significance level (alpha) was set to .05. The statistical analysis was performed using the R statistical package version 3.0.1 (the R Foundation for Statistical Computing, 2013).

## Results

### The Integration Framework

#### System Definition

The central element of SSM methodology is a real-world “problematic situation”. In our case, we define it as low level of interactivity of virtual patients to be increased through the integration of existing simulation technologies. We envisage a community where computational models produced by research projects are publicly available on the Internet and selected by the health care education community to extend the interactivity of existing or newly developed virtual patients.

The challenges of this approach are defined by different “worldviews” of the stakeholders (SSM Step 1) that include the biomedical modeling community, health care educators and learners, and virtual patient system developers.

The current perspective of the biomedical modeling community is well represented by the diversity of the VPH initiative. It focuses on the development and validation of patient-specific simulations to address clinical problems relevant to a particular patient population. Model construction is intellectually demanding with, as yet, little formalization. This focus is driven by researcher motivation, clinical drivers, and the availability of research funding, involving state-of-the-art technologies (eg, grid systems, cloud computing, supercomputers). One priority of the VPH initiative is to develop models and simulations that are able to provide a holistic view of the human body and able to deliver a technical infrastructure capable of providing access to different models and tools within a single computational workflow. Authoring of models and simulations is typically carried out in large international teams and requires formal consideration of ethical and intellectual property issues, particularly when industrial partners are involved. VPH researchers are largely unaware of the learning objectives of medical curricula, and the research focus of projects means that resources to adapt or maintain the tools for educational purposes are limited.

The worldviews of health care educators and learners, though clearly distinct, are closely interconnected and can be treated as one subsystem at the current stage of development of this framework. Driven by sound theoretical foundations, such as Kolb’s experiential learning theory [33], the community is building up an educational system where the learners experience the subject of interest by active experimentation, observations, and reflection. This group is willing to use simulations that clearly address learning objectives within the contemporary medical curriculum. It is important to consider factors such as the appropriate level of difficulty of the task and to promote high levels of interaction with the content or content-specific feedback [34]. However, caution is advised because too much interactivity may be detrimental to the learning process [35]. Most educators and students are not interested in, or do not have time, to study the technology that underpins the simulation. Their major expectation is to obtain a tool that is intuitive, works without delays, is well aligned with previous learning experience, and returns valid results. This group usually has access to standard IT equipment and Internet access, and neither the faculty nor the learners are willing to cover substantial additional expenses for access to simulation.

The worldview of the virtual patient system developer is, perhaps, not as clear to an outside observer. It is important to consider this perspective as the technical infrastructure in many medical universities has been in development for several years and is well established for educational workflows, particularly in the case of virtual patient systems [36]. Replacing well-functioning systems or adding new systems to an already complex e-learning infrastructure is likely to encounter resistance. These objections will be supported by health care educators who have already invested substantial resources in creating content for existing virtual patient systems. The virtual patient format, while existing in several variants, follows similar design rules [34,37]. In practice, this involves the presentation of a consistent story for a single patient, with new information unraveling over time. Virtual patient systems, which have been implemented in different technologies, have already achieved a common technological denominator in the form of the MVP standard [10].

#### Feasibility Features

The different perspectives characterized within the system definition were transformed into system features (Table 2). This list contains factors that should be considered when assessing the feasibility of simulation integration. To ensure that the most significant and timely factors have been included, its completeness has been validated against features identified in previous studies presenting the views of the three stakeholder groups (sources [9,11,16,34,37-41] are shown in the third column in Table 2). The list is not assumed to be exhaustive and has the potential to be extended in future.

**Table 2:** Features relevant for integrating computational models with virtual patients.

Stakeholder group	Identified features relevant for integrating computational models with virtual patients	Previous study
Computational modeling researchers	Availability of high-quality documentation (including a clear description of modeled parameters: their permitted input ranges, simulation steps, and post-processing steps)	[16,38,39]
	Validity of simulation results generated (compatibility with experimental data or expected observations)	
	Availability of model in machine-readable (preferably popular) format	
	Availability (and preferably mobility) of the simulation software for the model	
	Information on the magnitude of computational time required for simulation	
	Information on mobility and required storage space demands for input and output data, model, solver	
	Clearance of copyright issues (information about the authors of the model and terms of use and distribution)	
	Description of confidentiality constraints	
Health care education (educators and students)	Suitable learning objectives	[34,37,40]
	Relevance for study	
	Suitable target group	
	Appropriate level of difficulty	
	High interactivity	
	Availability of specific feedback	
	Optimal use of media	
	Focus of attention on relevant learning points	
	Recapitulation of key learning points	
	Authentic Web-based interface	
	Content tailored to the clinical reasoning process	
	Realistic narration to include the simulation in the case	
	Support for individualized approach to learning	
	Support for collaborative learning	
Virtual patient system developers	Simulation elements supported by the virtual patient system	[9,11,41]
	Simulation elements supported by the MVP standard	

#### Integration Levels

**Figure 2:**
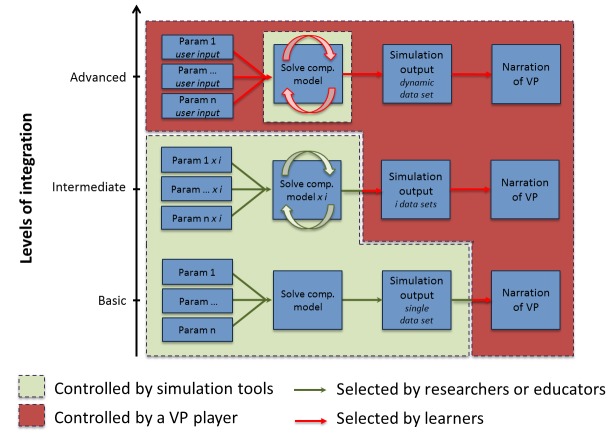
Three levels of integration of computational models into virtual patients.

Analysis of the feasibility features presented above depends largely on the extent to which the VPH-related data, models, or simulations are integrated within the virtual patient. This may be addressed by introducing different levels of integration. We propose three fundamental integration levels: (1) basic, (2) intermediate, and (3) advanced (Figure 2). In the discussion that follows, it is assumed that the virtual patient has a branching navigation model where the learner can select from a number of alternative options, solving the virtual patient with different possible end points [9]. The alternative linear model, where there is just one narration thread and one final end point, can be regarded as a special case of branching.

At the basic integration level, a single set of *n* input parameters is taken to generate a single outcome (or a single time-dependent outcome series) of biomedical variables for a single (virtual) patient. The results are reported within the narration of the virtual patient. For the intermediate level, pre-generation of simulation data over a range of input parameters is performed and included within the virtual patient package, for instance in the form of a look-up table. The execution environment enables the student to explore *i* different variants of data combination within the predefined constraints. The advanced level proposes a dynamic solution of the model while allowing the learner to work directly with the solver and freely specify the input within the domain of variables.

Figure 2 shows that, as the level of model integration is increased, the run-time control of the virtual patient simulation shifts from simulation infrastructure (green background in Figure 2) to the virtual patient player (red background in Figure 2). In the case of advanced integration, the model solver becomes an integral part of the virtual patient environment, enabling more self-reliance for the learner and, consequentially, supporting an explorative learning approach to a larger extent.

### Integration Strategies

#### Overview

Integration strategies provide guidance on how to implement the integration in practice. The basic level integration strategy is to manually copy the output of the solver to the relevant places in the virtual patient narrative (s_0_). In this paper, we propose four higher level strategies that apply to the intermediate and advanced levels of integration: (1) narrative integration, (2) integration with branching nodes, (3) characteristics of data, and (4) model location. The strategies are not mutually exclusive and can be applied in parallel. The list of integration strategies has the potential to be extended to encompass additional considerations in the future as both simulation and virtual patient technologies continue to develop.

#### Narrative Integration (s1)

This strategy determines how simulation outcomes fit within the narrative of the virtual patient. This is highlighted by the situation where a single patient cannot have different values of various anatomical or physiological parameters. Two modes of integration are proposed: “what-if-nodes” and “multiple-case-packages”. The what-if-node interrupts the narration of a single activity node in the navigation graph with a discovery learning task. Students are presented with a simulation output following the main narrative of the virtual patient but are encouraged to reflect how the results would differ if the input parameters change. By manipulating input parameter values (eg, by sliders or combo boxes), different simulation outcomes are loaded from a set of pre-generated values (intermediate integration level) or generated by dynamic solution of the model (advanced integration level). Learners can alter the input parameters an unlimited number of times. Unless combined with an integration mechanism that influences the branching nodes, after leaving the what-if-node the case resumes the original narrative thread of the virtual patient. One single case could encompass several what-if-nodes. The multiple-case-package entails dynamic creation of a whole population of virtual patients from one virtual patient package. The narrative of the activity nodes are formulated as templates with empty locations to hold values from either a set of pre-generated values (intermediate integration level) or dynamically generated by an integrated solver (advanced level). The selection of values for input parameters could be influenced by student interaction, follow a predefined range, or be selected at random and would not be changed after virtual patient navigation has started. The generation of these multiple cases would be carried out by the virtual patient player. A similar method has been used by Tworek et al in the Open Labyrinth virtual patient system to produce 97 virtual patients [42]. However, in this latter study the input values were taken from controlled vocabularies, statistical distributions of normal values, and manual correction of pathologies by subject experts and not dynamically created by solving computational models. Kononowicz et al proposed a variant of the multiple-case-package strategy for generating virtual patients from templates in computer-interpretable guidelines [32].

#### Integration With Branching Nodes (s2)

This strategy specifies whether simulated results have dynamic influence on branching nodes. In the “no influence” mode, the what-if-nodes and multiple-case-packages retain the same static branched navigation structure in all cases. No matter how the input parameter is manipulated in a what-if-node, after the student leaves this node the virtual patient player resumes the original narration thread. Similarly, a multiple-case-package would always have the same solution path. Alternatively, the definition of branching nodes could contain simulation variables encapsulated in a formal logical expression (influence on branching mode). These expressions would be evaluated dynamically by the virtual patient player during the run-time to automatically perform branching or to alter the scoring of decisions made by the student while solving the case. In this mode, the what-if-node would change the succeeding activity sub-trees. A potential scenario might involve a student trying out different levels of drug dosage to discover immediate reactions of the virtual patient before making the final decision. For the multiple-case-package, the same route taken by the student through the activity graph could result in different scoring based on the simulated output of a randomized or pre-selected input set.

#### Characteristics of Data (s3)

This strategy describes the selection of model input parameters. The “simple data” mode allows selection of any combination of values from permitted input ranges. At the intermediate level of model integration, these ranges are discrete whereas, for the advanced level, they might be close to continuous. The designer of the virtual patient may wish to include input parameters that do not influence simulation outcomes to improve student motivation, include distractors as part of the learning design or increase the realism of the case. Examples of distractor parameters include the name of the virtual patient, description of the hospital setting, or physiological parameters that do not influence the simulation (eg, eye color) [42]. The “simple data with exclusion” level enables specification of a set of excluded input values (to define a subspace of the permitted input space) for which no simulation results will be generated (eg, because they are physiologically impossible). Finally, for “interdependent data” a functional connection (eg, gender specific values, values with a specific non-negligible biological feedback loop, dependencies preserving anatomical continuity, etc) would be defined between some input variables to either guide or restrict the learner’s choice of input parameters.

#### Model Location (s4)

This strategy defines where simulated data or simulation software is located. For the intermediate level of integration operating in a “local” mode, the generated data are located within the virtual patient package. A “distant” mode would involve data dynamically loaded from a central repository of pre-generated data (because of substantial storage space requirements or for confidentiality due to sensitive, patient-specific information, for instance [43]). This could be managed either by the virtual patient system itself or through a service independently accessible on the Internet. For the advanced level of integration, where model solution is integrated with virtual patient navigation, the local mode requires either direct incorporation of the solver within the virtual patient package or inclusion of the solver as part of virtual patient execution environment. The advanced level of integration operating in a distant mode requires communication with an external solver service, for example, by Web Services or some other form of Web interface.

#### Evaluation Profiles

**Figure 3:**
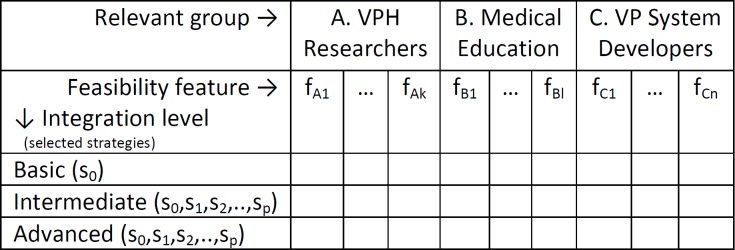
Method for systematic evaluation of different levels of simulation outcome integration.

Decisions about the feasibility and/or desirability of performing a particular level of integration for a given computational model should be made after considering the perspectives of the relevant stakeholders. This is represented visually in Figure 3 and forms a tool for evaluation of the feasibility of integration (SSM Step 2). The columns represent the features identified in Table 2 for particular stakeholder groups, identified by the notation f_[{A,B,C}]_, where the subscripts define the stakeholder group and the feature reference number. The integration levels are also parameterized by the integration strategies (s_0_,s_1_,s_2_,...,s_p_).

The evaluation table can be populated through the use of evaluation profiles to guide consultation with the appropriate stakeholder communities. An evaluation profile is defined by the selection of a subset of feasibility features and specification of acceptance thresholds for critical features to reflect the priorities and requirements of the integration. These evaluation profiles can be used to determine the status of cells using colors (green—threshold passed; red—failed; yellow—borderline or unknown; grey—not relevant for decision). If a threshold is not specified for a given feasibility feature, the feature may be discussed during consultation to provide qualitative feedback, but not quantitatively evaluated. Potential evaluation profiles classes might include “integration for optimal exposure of the VPH tool” (ie, project dissemination purpose), “integration for high interactivity”, “integration for high relevance to formative assessment within a particular curriculum”, and “integration for optimal use in a particular virtual patient environment” (eg, in “Open Labyrinth” virtual patient player).

### Demonstration of the Framework on a Real-World Example

#### Profile

An evaluation profile of the type “integration for optimal exposure of the VPH tool” was defined for the archTk simulation (SSM Step 3). Tables 3 and 4 present details of the profile including feasibility features, their acceptable thresholds (Table 3), and selected integration strategy modes (Table 4). The designated thresholds reflect the requirements for presenting the project outcomes in workshops and postgraduate courses using standard PCs or tablets without significant computational delays (<5 seconds).

**Table 3:** VPH ARCH evaluation profile—selected feasibility factors and thresholds.

A. VPH researchers	AT^1^	B. Medical education	AT	C. VP system developers	AT
f_A1_: computational time	<5s^2^	f_B1_: suitable target group	Any^3^	f_C1_: VP player support	–
f_A2_: storage requirements	(<1MB)	f_B2_: appropriate level of difficulty	–	f_C2_: MVP standard support	–
f_A3_: results validity	^4^				
f_A4_: intellectual property	Yes				

^1^AT: acceptance threshold.

^2^Time measured by the student.

^3^Within the context of health professionals.

^4^Face validity (system performs as a subject matter expert would intuitively expect).

**Table 4:** VPH ARCH evaluation profile—selected integration strategies.

Strategy	
s_1_: narration integration	(one) what-if-node
s_2_: integration with branching nodes	No influence
s_3_: characteristics of data	Changeable input parameters: gender, age, height, weight
	Output parameters: Predicted brachial artery flow (mL/min) and pressure (mmHg)
	Dependent variables: weight and height values specific to gender, typical distributions of vascular anatomy.
s_4_: model location	Local (within virtual patient player)

#### Computational Modeling Researchers Feasibility Features

The pulse wave propagation model applied in the VPH ARCH project as part of the archTk toolkit was initially developed by Huberts et al [28]. It depends on over 70 parameters, many of which are patient-specific and difficult to measure [44]. To decrease model complexity, the number of parameters may be reduced by generating typical distributions of vascular anatomy and material properties for a male and female of specific age and BMI. In the virtual patient context, the input parameters are expressed as weight, height, gender, and age. In general, the patient-specific nature of such computational models complicates the use of generic parameter sets. However, the use of this approach for the archTk model is supported by published data [45]. Assessment of the influence of the choice of input parameters on the effectiveness of model integration has not been undertaken in this study and is an important topic for further refinement of the framework.

Generation of a single set of output values on a standard PC workstation (Intel Core 2; 1.66GHz; 4GB RAM) for the archTk model takes 5 minutes. At the intermediate level of integration, we have evaluated a test case with a range of input parameters that include two values for the gender parameter and two values of age, weight, and height, which depend on gender, giving a total of 16 result sets. Calculation of this level of simulation data requires 80 minutes of computation prior to virtual patient navigation. This is feasible. The display of pre-generated values takes less than one second, which is sufficiently short to ensure acceptable results at the basic and intermediate level of integration. Dynamic generation of a dataset is not efficient enough on a standard PC with the current solver implementation (pyNS 0.4.2) to support a swift (<5s) response of the graphical user interface. Storage requirements for all levels of integration are negligible as the model output consists of small numbers of ASCII files.

We were able to test the validity of the outcomes for the basic and intermediate level of integration with the experts with whom we are collaborating. The validity of the advanced level of integration cannot be assessed with the current level of development of the simulation software.

Intellectual property issues are not a barrier with this application. The software is available in an open-source form allowing unrestricted use for educational purposes.

#### Health Care Education Feasibility Features

An evaluation was performed of the integration potential of the VPH ARCH project virtual patient before initiating the study through collaboration with AMEE representatives. A challenge was identified during this consultation relating to the exploitation of such simulations for undergraduate level training. The high level of clinical specialization of the content exceeds the scope of the undergraduate curriculum; vascular access surgeon training was recommended as a more suitable target. A second target group identified was medical science or medical informatics students with an interest in the application of information and communications technology methods in medicine.

#### Virtual Patient System Developers Feasibility Features

Presentation of simulation data at the basic level of integration of the archTk is possible in any virtual patient system compatible with the MVP standard. The what-if-node is not currently supported by many virtual patient systems or by the MVP standard, and while it is possible to generate 18 activity nodes to represent each what-if node, this process is cumbersome for the end user and could result in practical limitations for large input parameter ranges. We do not regard the current status of the f_C1_feature (virtual patient player support) as critical since we intend to extend existing open-source virtual patient systems to accommodate such features on which the intermediate level of integration relies.

**Figure 4:**
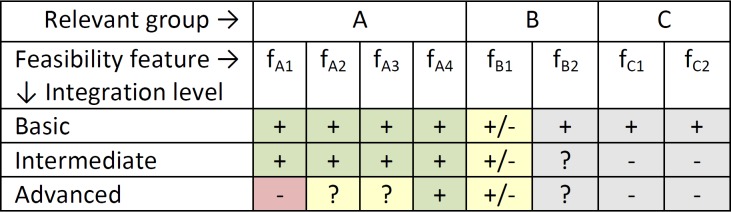
Evaluation profile for the VPH ARCH integration.

The feasibility analysis of the VPH ARCH simulation integration for “integration for optimal exposure of the VPH tool” profile (Figure 4) concluded that integration is possible for the basic and intermediate levels.

### Implementation of Two Levels of Integration

**Figure 5:**
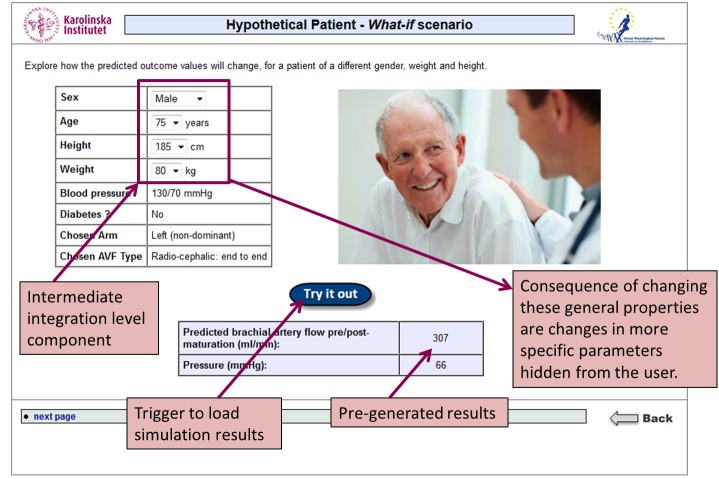
Second level computational model integration ("what-if-node") for the VPH ARCH project virtual patient.

Following the positive evaluation of the integration potential of the VPH ARCH project at the basic and intermediate levels, two variants of the same virtual patient were implemented reflecting these levels. The first case (Study Group 1, Basic Integration Level) presented static simulation results through manual pasting into the case (s_0_ integration strategy), whereas the second case (Study Group 2, Intermediate Integration Level) provided the user with the option to manipulate different parameters of the simulation. The narrative integration (s_1_) was implemented by adding a what-if-node at the end of the case (Figure 5).

Users of the second group could not circumvent the opportunity to use this feature as it was placed in the central navigation path. There was no influence of the parameter manipulation on the virtual patient branching (s_2_). The input parameters of gender, age, height, and weight are interdependent and represent a set of input parameters with typical distributions of vascular anatomy for a male and female of specific age and Body Mass Index (s_3_). The simulation data (16 result sets) were generated prior to the experiment on a standard PC workstation (Intel Core 2; 1.66GHz; 4GB RAM), at run-time loaded from a pre-generated look-up table located in the virtual patient player (deployed at a WWW server) and available locally for manipulation in the Web browser through a JavaScript method (s_4_).

### Perception of the Framework Output by Health Care Workers

Table 5 summarizes the responses of the participants at the EVC conference event. The virtual patient was interesting or very interesting for 84% of participants (32/38), 11% (4/38) were neutral, and 5% (2/38) found the virtual patient “not that interesting” or “not at all interesting”. The average evaluation score (Likert scale, 5=very interesting, 1=not at all interesting) was better for the intermediate integration level (4.22) than the basic level (4.10) in this sample, but the difference was not statistically significant (*P*=.84). None of the evaluators disagreed or strongly disagreed with the presented content, 76% (29/38) agreed or strongly agreed with it, and 24% (9/38) neither agreed nor disagreed. The average evaluation score (Likert scale, 5=strongly agree, 1=strongly disagree) was better in this case for the basic integration level (4.05) than the intermediate level (3.83). Again this difference was not statistically significant (*P*=.32).

In the questions directed to Study Group 2 only, “The possibility of manipulating simulation parameters (gender, age, weight, height) for the hypothetical patient was …”, 28% (5/18) responded with “very interesting”, 39% (7/18) “interesting”, 33% (6/18) “neutral”, and no one answered “not that”, “not at all interesting”. It should be stressed that no participants responded “I have not noticed this possibility” to this question, which was provided as an alternative option. The participants in Study Group 2 were also largely satisfied with the results of manipulating the simulation parameters. To the question “Did the results of manipulating the simulation parameters meet your expectations?”, 11% (2/18) answered “strongly agree”, 72% (13/18) “agree”, and 17% (3/18) “neither agree nor disagree”. No one disagreed with this statement or had not used this function.

**Table 5:** Summary of answers in the evaluation questionnaire.

Question	Likert scale	Group 1 n=20	Group 2 n=18	*P*
Was the presented virtual patient case interesting for you?	5=very interesting; 1=not at all interesting	4.10	4.22	.84
Do you agree with the presented content of the virtual patient case?	5=strongly agree; 1=strongly disagree	4.05	3.83	.32

## Discussion

### Summary

Integration of simulation is gaining importance in health care education informatics. After a period where isolated systems were developed, the advantages of combining systems into “continua of simulation” have become apparent [15]. This connection not only augments the value of learning activities by facilitating wider opportunities to synthesize knowledge and skills, but it also fosters reuse of existing models, thereby increasing the return on investment in their development.

We may regard the proposed framework as a specialization of a section of Ellaway’s et al “Practica continua” framework [15]. Some integration strategies we have proposed are direct responses to the desiderata of this framework. For instance, our narrative integration strategy (s_1_) has a clear correspondence to the “narrative integration” dimension and requirements (eg, timeline and causality continua) in Ellaway et al. The added value is that we have exemplified their implementation in the context of virtual patients and computational models of physiology by concrete implementation proposals in the form of the “what-if-node” and “multi-case-package” strategies. On the other hand, our framework is not just a specialization of the “Practica continua” framework, since it discusses the integration of computational models that are products of research projects initially outside the educational sector. This consideration is given in our framework by inclusion of tools for systematic evaluation of the feasibility of integration. The framework described here is currently restricted to the integration activity phases, that is, the authoring process. This leaves the run-time and analysis phases for further iterations of framework development. The HSVO project, related to the “Practica continua” framework, demonstrated how the run-time phase may be implemented by a common execution interface and middleware layer [46]. These outcomes will be useful in the further development of our framework.

It is important to stress that current virtual patient systems and the MVP standard are not yet, in general, ready for the higher levels of integration described in this paper. However, the effort involved in extending virtual patient systems to facilitate the intermediate level of model integration does not seem to be obstructive. A graphical user interface component to handle the what-if-node manipulation of model parameters and display the simulation results is of primary importance along with a standard mechanism to manage pre-generated data in virtual patient packages. The advanced level of integration poses a more significant challenge. Many models available today have been implemented using scientific tools such as MatLab (Mathworks) or specific numerical libraries. This limits their portability in virtual patient packages. A viable strategy might be to host the solver on a remote server, but this conflicts with the self-containment rule of content of a virtual patient package, raises security concerns, and requires the maintenance of an additional service. These considerations will fuel further research as suggested by this paper (SSM Step 4).

The proposal of this framework was helpful for the authors in developing, discussing, and reporting the integration of the archTk simulation within the virtual patient. It is challenging to demonstrate, in the short term, the long-term benefits of the framework for the users of virtual patients. The evaluation study carried out at the EVC conference provided an unambiguously positive response to the approach of integrating advanced computational models of physiological processes within virtual patients. Based on the obtained feedback sample, we hypothesize an altered perception of integration depending on changes in the framework’s variables. The queried group of experts found higher integration more interesting while, at the same time, being more concerned with the validity of the results. It has to be stressed, however, that this conclusion cannot be generalized as yet because the difference is not statistically significant and the influences of other factors (eg, age or experience) could not be excluded.

### Limitations

The application of the integration framework has been demonstrated by a case study involving a single computational model. Improvements for the next SSM learning cycle should aim to extend the proposed lists of feasibility features, integration strategies and scope of exemplar projects. This cycle may be repeated several times depending on the research outcomes, prioritizing different simulation aspects.

Some proposed integration modes are currently theoretical constructs that require testing on concrete examples. In particular, this applies to the advanced level of integration that was not attempted in this case because the computational load was unsuitable for target application. Future SSM iterations should focus on concrete data representations and software solutions enabling further development of the integration strategies and evaluation process.

The educational impact of the proposed integration levels is still uncertain and needs to be tested in rigorously conducted comparative studies. The integration of the archTk simulation into a virtual patient was tested with clinical experts and health care education specialists, but not on a wider scale with different virtual patient systems. The completeness and priority of the proposed framework elements could be addressed by a Delphi study to formally collect feedback from computational modeling researchers and virtual patient system developers.

### Conclusions

The paper has outlined a conceptual framework for the integration of computational models into virtual patients. This includes consideration of feasibility features, levels of integration, integration strategies with various modes, and evaluation profiles. The opportunities and challenges of model exploitation have been discussed in the context of a virtual patient developed from the VPH ARCH project, incorporating archTk simulation results at different integration levels. The empirical evaluation of two variants of the virtual patient provided positive feedback on the value of this type of integration. The responses suggest further investigation of increased user satisfaction, but decreased trust, at higher levels of simulation integration. The long-term research aim is to isolate the most crucial factors in the integration framework and their influence on the integration outcome.

## Abbreviations

AVF: arteriovenous fistula
ESRD: end-stage renal disease
EVC: European Vascular Course
MVP: MedBiquitous Virtual Patient Standard
NoE: Network of Excellence
SSM: Soft System Methodology
VP: virtual patient
VPH: Virtual Physiological Human
